# Differences in the antibody response to adult *Fasciola hepatica* excretory/secretory products in experimentally and naturally infected cattle and sheep

**DOI:** 10.1016/j.vetpar.2020.109321

**Published:** 2021-01

**Authors:** Tessa R Walsh, Stuart Ainsworth, Stuart Armstrong, Jane Hodgkinson, Diana Williams

**Affiliations:** Department of Infection Biology and Microbiomes, Institute of Infection, Veterinary and Ecological Sciences, University of Liverpool, L69 7ZX, UK

**Keywords:** FEC, Faecal egg count, wpi, Weeks Post Infection, ES, Excretory/Secretory, CL, Cathespin, rCL1, Recombinant cathepsin L1, RPMI, Roswell Park Memorial Institute, YPDS, Yeast-Extract, Peptone, Dextrose, Sorbitol, rpm, Revolutions per minute, OD, Optical density, BGMY, Buffered Glycerol Complex Medium, BMMY, Buffered Methanol Complex Medium, MWCO, molecular weight cut off, PVDF, Immobilon-P membrane polyvinylidene fluoride, DAB, 3′-Diaminobenzidene, HRP, Horse raddish peroxidase, TMB, 3,3′,5,5′-Tetramethylbenzidine, TCA, Trichloroacetic acid, DTT, Dithiothreitol, IAA, Iodoacetamide, TFA, Trifluoracetic acid, GST, Glutathione S transferase, DLD, dihydrolipoyl dehydrogenase, *Fasciola hepatica*, Liver fluke, Cathepsin L1, Serodiagnosis, Antibody response

## Abstract

*Fasciola hepatica* (the liver fluke) is a common, global parasite of livestock. It can be highly pathogenic and has health and welfare implications for infected individuals. Typically, in ruminants, infections are sub-clinical, but if undiagnosed, they can lead to significant production losses. Accurate diagnosis is crucial to identify infection. Antibody detection ELISAs are commonly used to diagnose infection due to their high sensitivity and specificity and are typically based on native fluke excretory/secretory (ES) products or cathepsin L1 (CL1), the immunodominant antigen within ES products. These tests have been developed based on the antibody response of experimentally infected animals; however, this response has not been well characterised in naturally infected animals. We compared the antibody recognition of a recombinant CL1 (rCL1) antigen and native adult fluke ES products. Whilst samples from experimentally infected animals showed strong recognition of rCL1, serum antibodies from naturally infected animals did not. These results were confirmed by peptide array. Immunoblotting sera against ES products showed that experimentally infected animals had a strong, specific response to CL1/CL2 proteins whilst antibodies from naturally infected animals recognised multiple proteins and had a variable response to CL1/CL2. Mass spectrometry of proteins separated by 2D SDS PAGE, identified several antigens recognised by serum antibodies from a naturally infected cow, including cathepsins L1, L2 and L5, glutathione S-transferase and a dihydrolipoyl dehydrogenase. Overall, these results show that the antibody response in naturally infected animals to adult fluke ES products is qualitatively different to experimentally infected animals. This suggests that a diagnostic test based on CL1 alone may not be appropriate for diagnosis of natural *F. hepatica* infections in sheep and cattle.

## Highlights

• Antibody response is different in animals experimentally and naturally infected with *F. hepatica*.
• Experimentally infected animals specifically recognised cathepsin proteins.
• Naturally infected animals showed poor recognition of a recombinant cathepsin L1.
• Antibody response of naturally infected animals is against multiple antigens.
• Diagnostic tests based on a single antigen may not be suitable for use in field.

## 1 Introduction

*Fasciola hepatica* (the liver fluke) is a common parasite with a worldwide distribution, typically found in areas with temperate climates. It is one of the causative agents of fasciolosis, affecting a wide range of host species. In the UK, *F. hepatica* is a major pathogen of ruminant livestock. Infections with *F. hepatica* result in significant consequences for the health and welfare of the animal. Chronic infections result in anaemia, weight loss and ill-thrift. Acute infections can be fatal as a result of substantial liver damage caused by migration of large numbers of juvenile fluke. Low grade infections are common, sub-clinical and are associated with reduced growth rates and decreased milk yield (Charlier et al., 2007; Mezo et al., 2011; Charlier et al., 2012; Howell et al., 2015; Köstenberger et al., 2017; Mazeri et al., 2017). With prevalence of fluke predicted to rise due to climate change (Fox et al., 2011; Caminade et al., 2015) and the increasing prevalence of resistance to triclabendazole (Kamaludeen et al., 2019), strategic control programmes that rely less on repeated whole herd or flock drug treatment programmes are needed. Accurate diagnosis of infection is a vital component of such programmes.

Traditionally, *F. hepatica* infections are diagnosed using faecal egg counts (FEC). FEC are simple to perform but have a low sensitivity, particularly in cattle (30–70 %) and can only detect patent infection (Charlier et al., 2014). Other methods for diagnosing fluke infection include antibody detection ELISAs that detect antibodies against fluke at 2–4 weeks post infection (wpi) in serum and milk samples (Salimi-Bejestani et al., 2005, 2007). These ELISAs are typically based on native fluke excretory/secretory (ES) products due to their strong immunogenic properties (Alvarez Rojas et al., 2014). Whilst these tests tend to have a high sensitivity (86.1–100 %) and specificity (70–99.3 %) (Cornelissen et al., 1999; Salimi-Bejestani et al., 2005; Kuerpick et al., 2013; Gottstein et al., 2014) the use of native ES products in commercial diagnostic tests is not ideal as they rely on a regular supply of live adult fluke and extensive evaluation is needed to ensure consistency between each preparation of the antigen.

ES products are a complex mixture, with proteases accounting for 73 % of the total protein content including cathepsin L, cathepsin B, metalloproteases, serinoprotenases and legumains (Di Maggio et al., 2016). Other proteins can be released from blebbing or shedding of the flukes tegumental surface during culture, and may also be present in ES preparations (Robinson et al., 2009; La Course et al., 2012).

Early studies showed that experimentally infected animals exhibit a strong antibody response to proteins between 24−26k Da in adult fluke ES products (Alvarez Rojas et al., 2014) which were later identified as cathepsin L1 (CL1) and cathepsin L2 (CL2) (Dalton and Heffernan, 1989; Smith et al., 1993a; Dowd et al., 1994). CL1 is the most abundant of the two proteins, accounting for 67 % of total cathepsin L proteins compared to 28 % for CL2 (Robinson et al., 2008). CL1 and CL2 play a key part in the pathogencity of the fluke, being able to cleave haemoglobin, degrade structural proteins such as collagen, fibronectin and laminin, and degrade host IgG molecules (Berasaín et al., 1997; Smith et al., 1993b; Stack et al., 2008; Lowther et al., 2009).

Due to its immunodominant properties, CL1 has been the antigen of choice for diagnostic tests and as a potential vaccine candidate (Dalton et al., 1996; Alvarez Rojas et al., 2014). Recombinant CL1s (rCL1) have been described in multiple studies (Roche et al., 1997; Cornelissen et al., 2001; Collins et al., 2004; Stack et al., 2008; Kuerpick et al., 2013) and diagnostic ELISAs based on different rCL1s have reported high sensitivity (90–100 %) and specificity (88.6–98.5 %) (Cornelissen et al., 2001; Kuerpick et al., 2013).

Sera from experimentally infected animals have been used to evaluate these diagnostic rCL1 ELISAs (Cornelissen et al., 2001; Kuerpick et al., 2013), however the results were not directly compared to ES ELISAs (Cornelissen et al., 2001). Experimentally infected animals are normally exposed to a single, large bolus of metacercariae and then euthanized or treated after a relatively short period. There are few studies which have investigated antibody responses in naturally infected animals (Gorman et al., 1997; Ortiz et al., 2013). Natural infections are normally chronic, lasting months or years and associated with repeated exposure. The antibody repertoire in naturally chronically infected cattle and sheep has not been well characterised and it is unclear if it is similar to that seen in experimentally infected animals early in infection (12–20 wpi). In this study, we show that antibody responses in naturally infected cattle and sheep are qualitatively different to those of experimentally infected animals, and that not all naturally infected animals have a consistent detectable antibody response to either native or recombinant CL1. This could influence the choice of antigens to include in future immunodiagnostic tests and suggests that a test relying on a single antigen may be inappropriate in a commercial diagnostic test.

## 2 Materials and methods

### 2.1 Preparation of fluke antigens

Fluke infected sheep livers were collected from a local abattoir within 2 h of slaughter and adult fluke isolated from bile ducts. Fluke were washed in pre-warmed PBS (Cell-culture grade, Sigma Aldrich, UK) and then placed individually into 1 mL of sterile Roswell Park Memorial Institute (RPMI) media (Sigma Aldrich) containing 1% penicillin/streptomycin for 2 h to purge caecal contents. Fluke were washed again in PBS and live fluke were placed in groups of 10–15 in 25 cm^2^ Corning tissue culture flasks (Appleton Woods, UK) in 5 mL RPMI media overnight at 37 °C. Fluke were examined for movement and presence of eggs to confirm viability at the end of the incubation period (∼14 h). Media from flasks containing live fluke was pooled and eggs and debris removed by centrifugation for 20 min at 600 *xg* at 4 °C. The supernatant was passed through a 0.2 μm filter and frozen at −80 °C in 1 mL aliquots until required. Total protein concentration was measured using the Bradford Assay (Bradford, 1976).

### 2.2 Preparation of CL1 cDNA from adult fluke tissue

RNA was extracted from a single archived adult fluke obtained from the liver of a naturally infected sheep (sourced from an abattoir in central England), with the approximate time from slaughter to fluke extraction being 6−8 h. The fluke were incubated in Dulbecco’s Modified Eagle’s Media (Sigma-Aldrich) with 120 μg/mL of gentamicin (Sigma-Aldrich) and 120 μg/mL of amphotericin B (Sigma-Aldrich) for 2 h to purge eggs. The fluke were then snap frozen on dry ice and stored at −80 °C. Half the fluke was homogenised in 1 mL TRIzol solution (Invitrogen, UK) following manufacturer’s protocol. The RNA pellet was air dried for 15 min at room temperature and resuspended in 100 μL of RNAase free water (Ambion, USA) by incubation at 55 °C for 10 min. The sample was stored at −80 °C until needed. RNA concentration was quantified using Ribogreen (Life Technologies, UK). Qiagen QuantiTect Reverse Transcription Kit (Qiagen, UK) was used to convert 1 μg of RNA to cDNA using manufacturer’s instructions.

### 2.3 pPinkα-HC-CL1 expression vector construction

The CL1 gene was amplified from 1 μg of fluke cDNA using Phusion Green Hot Start II High-Fidelity PCR Master Mix (Thermo Scientific, UK). Primers were designed using CL1 sequence U62288 (NCBI Genbank) (Roche et al., 1997) as per manufacturer’s requirement’s for successful cloning and expression in PichiaPink Expression System (Invitrogen, UK), (CL1-F, 5′-CACCAGCAGCAGGCCTCGAGACAGAACGTCCGAT-‘3 and CL1-R, 5′-AGCAGCGGTACCTCAGTGGTGGTGGTGGTGGTGCGGAAATCGTGCCACCAT-‘3). CL1 amplification cycling conditions were 30 s at 98 °C, followed by 35 cycles of 98 °C for 10 s, 65.1 °C for 30 s, 72 °C for 45 s and 10 min at 72 °C. PCR products were sequenced by Sanger sequencing (Source Biosciences, UK) to confirm correct amplification of CL1 gene.

Amplified CL1 was cloned into pENTR vector (pENTR-CL1) using Topo cloning as per manufacturer protocols. This plasmid was maintained in *Escherichia coli* One Shot® TOP10 (Invitrogen, UK), with Sanger sequencing (Source Bioscience) performed to confirm gene integrity. CL1 was subsequently amplified (as above) from pENTR-CL1 and restricted with StuI and KpnI (Invitrogen) prior to ligation into similarly restricted pPinkα−HC vector (pPinkα−HC-CL1). Ligated plasmids were transformed into *E. coli* One Shot® TOP10 for screening, sequencing and maintenance of correctly constructed pPinkα−HC-CL1.

### 2.4 Recombinant CL1 expression

pPinkα−HC-CL1 was linerized for transformation into *Picha pastoris* (strain 2, Invitrogen) with XagNI (EcoNI) (Invitrogen) as per manufacturer’s protocols. Electrocompent *P. pastoris* were prepared as per manufacturer’s protocols and used immediately for transformation. Briefly, 80 μL of electrocompetent *P. pastoris* cells were mixed with 5 μL of linearized pPinkα−HC-CL1 vector and transferred to an ice-cold 0.1 cm electroporation cuvette (Geneflow, UK). This was incubated for 5 min on ice prior to electroporation using a Thermo EC electroporator at 1800 V, which was followed immediately by the addition of 1 mL of ice-cold Yeast-Extract, Peptone, Dextrose, Sorbitol (YPDS) media. Cells were incubated at 30 °C without shaking to recover for approximately 4 h prior to plating on selective Pichia Adenine Dropout (PAD) agar plates. Plates were incubated at 30 °C for at least seven days to allow the appearance of distinct colonies. Successfully transformed pPinkα−HC-CL1 *P. pastoris* colonies were identified via PCR using vector specific (AOX1/CYC1 [Invitrogen]) and insert specific (CL1-F/CL1-R) primers.

Expression of rCL1 was performed using the Picha Pink expression kit (Invitrogen) as per manufacturer’s instructions with modifications. Individual *P. pastoris* pPinkα−HC-CL1 colonies were inoculated into 25 mL of Buffered Glycerol Complex Medium (BGMY), adjusted to pH 8.0 (Collins et al., 2004) and incubated for 24 h at 30 °C with shaking (300 revolutions per minute [rpm]) until the culture had reached an optical density (OD)_600_ of 2−6. This seed culture was inoculated into 1 L of BGMY (pH 8.0) and incubated as above for 24 h, until the culture reached an OD_600_ of 2−6. Cells were harvested by centrifugation (5 min, 1500 *xg*) and resuspended in 200 mL of the expression medium; Buffered Methanol Complex Medium (BMMY) (pH 8.0). Cultures were maintained for four days at 23.5 °C (Li et al., 2001) with shaking (300 rpm). For continual rCL1 expression, filter sterilised methanol was added every 24 h to maintain a methanol concentration of 1% in culture (Collins et al., 2004). After four days cells were harvested by centrifugation (as above) and the supernatant collected and concentrated (Amicon Ultra centrifugal filters, 10 kDa molecular weight cut off [MWCO]). If rCL1 was not purified immediately, concentrated supernatant was stored at 4 °C.

rCL1 protein was purified from concentrated culture supernatants by affinity chromatography. The culture supernatant was mixed with binding buffer (50 mM sodium monophosphate [pH 8.0], 300 mM NaCl and 10 mM imidazole) at a 1:1 ratio and run over 1 mL of Ni-NTA agarose bead slurry (Qiagen) under gravity flow using disposable chromatography columns (BioRad). The agarose beads were washed with 25 mL of wash buffer (sodium monophosphate [pH 8.0] containing 50 mM sodium monophosphate, 300 mM NaCl and 20 mM imidazole) prior to rCL1 elution with 5 x 2.5 mL of elution buffer (50 mM sodium monophosphate [pH 7.0], 300 mM NaCl and 250 mM imidazole). Purity of rCL1 was determined using SDS PAGE and Western Blot as described below. Fractions harbouring rCL1 were pooled and buffer exchanged into PBS (Amicon Ultra 4 mL [MWCO 10 kDa]) or dialysed vs. PBS (7 kDa MWCO Slide-A-Lyzer [Thermo Scientific, UK]) overnight at 4 °C. Purified rCL1 was aliquoted and stored at −20 °C.

### 2.5 Serum samples

Archived serum samples collected from naturally and experimentally infected cattle and sheep were used. Samples were aliquoted as soon as possible after collection and stored at −20 °C until use. Samples were collected from experimentally infected sheep (N = 12, 0–14 wpi) and cattle (N = 4, 0–14 wpi). Sheep and cattle were experimentally infected with 200 and 1000 metacercariae respectively. Blood samples were collected from naturally infected cattle and sheep on farms with a history of fluke infection, confirmed by diagnostic assays or abattoir returns. Serum was obtained from adult dairy cattle (N = 11) at drying off, and adult breeding sheep (N = 19). All animals had been grazed for at least one season or more on fluke infected pasture. Sera was also collected monthly from calves (N = 10) grazed on fluke infected pasture over their first-year grazing season. Individual faecal samples were also collected at time of sampling to confirm infection by FEC for all animals.

The positive and negative control sera used in the ES and rCL1 ELISAs, immunoblotting and peptide arrays were as follows: positive cattle control were made from a pool of sera taken from naturally infected cattle with FEC confirmed infection; positive sheep control was taken from an experimentally infected sheep with chronic infection, confirmed through FEC. The negative control sera was from a cow and a sheep, born and kept indoors at the University of Liverpool farm where there is no evidence of fluke infection.

### 2.6 ELISA

Serum samples were tested in duplicate. OD values were averaged and expressed as a percentage of the positive control (percent positivity [PP]) using the following formula: PP = (mean OD of test sample/mean OD of positive control) x100. An arbitrary cut-off of 14 PP was calculated by ROC analysis using data from experimentally infected sheep for the sheep ES ELISA, (sensitivity 95 %, specificity 100 %, [Walsh, 2018]). A diagnostic cut-off of 15 PP was used for cattle serum samples (sensitivity 98 %, specificity 96 %) (Salimi-Bejestani et al., 2005).

Serum samples were tested in the ES ELISA and in the rCL1 ELISA. Samples for experimentally infected animals consisted of week zero (pre-infection), week four (early-infection), and week nine for sheep or week 11 for cattle (late-infection). For naturally infected calves, time points tested by rCL1 ELISA were before turnout (Month 0) and at six months after turnout or the closest time point thereafter as calves had seroconverted by this time-point (Graham-Brown et al., 2018).

The ELISA protocols were based on methods described by Salimi-Bejestani et al. (2005). For cattle rCL1 ELISA, plates were coated at 0.5 μg/mL. For the sheep ELISA, plates were coated with either 0.5 μg/mL of ES products or 0.1 μg/mL of rCL1 and the conjugate was a monoclonal anti-goat/sheep IgG-peroxidase antibody (Clone GT-34, Sigma Aldrich).

### 2.7 Peptide array

Peptide arrays were synthesised by JPT Peptide Technologies (Berlin, Germany, PepStar peptide microarray service). The rCL1 protein sequence was divided into 15 amino acid long peptides (N = 82), each with a 12 residue overlap to the preceding peptide. Each peptide was covalently immobilised onto glass slides via a hydrophilic linker molecule, arranged into blocks, each containing three-sub arrays.

Serum samples from experimentally and naturally infected animals were tested in the arrays. For samples from naturally infected animals, sera showed variable recognition to the rCL1 antigen. Therefore, for peptide arrays, sera with a range of PP values (high, medium or low) on the rCL1 ELISA were chosen.

Sera were diluted 1:200 in array blocking buffer (Superblock TBS T20, Pierce International) and applied individually to each array block. Slides were incubated at 30 °C for 1 h before washed in washing buffer (50 mM TBS-buffer, 0.1 % Tween20 pH 7.2). Slides were incubated for 1 h with the secondary antibody at 30 °C. For cattle samples, the secondary antibody was unconjugated monoclonal anti-bovine IgG produced in mouse (clone BG-18, Sigma Aldrich) diluted at 1:1000 in array blocking buffer. For sheep samples, unconjugated monoclonal mouse anti sheep/goat IgG (Clone GT-34, Sigma Aldrich) was used at 1:5000 in array blocking buffer. Slides were washed and then incubated at 30 °C with a tertiary fluorescent antibody (anti-mouse, IgG [H&L] DyLight 650, Thermo Scientific) at 0.1 μg/mL. Slides were washed for a final time and dried before antibody binding was visualised by Axon Genepix Scanner 4300A (Axon Instruments) at 635 nm to obtain fluorescence intensity profiles for each spot. Resulting images were quantified and the mean pixel value for each of the three sub-arrays was averaged for each peptide and serum sample using GenePix (Molecular Devices).

### 2.8 Crystal 3D model of CL1

A 3D model of CL1 was generated using SWISS-MODEL (Arnold et al., 2006), based on a published structure (Stack et al., 2008). The model was visualised and annotated using DeepView/SWISS-Pdb Viewer (Version 4.1.0) (Guex et al., 2009).

### 2.9 Immuno/Western blotting

ES products (100 ug/mL) were separated on 12 % reducing SDS PAGE gels alongside a molecular weight marker (Broad Range ladder, NEB, UK) prior to transfer onto activated Immobilon-P membrane polyvinylidene fluoride (PVDF) membranes, 0.45 μm (Sigma Aldrich, UK) by wet Western transfer method. Membranes were blocked for 1 h in Western blocking buffer (4% skimmed milk powder [Marvel] in PBS) at room temperature and then washed three times for 15 min each in PBS Tween (PBST, 0.05 % Tween). Membranes were cut into strips and incubated individually with cattle or sheep serum at 1:200 dilution in Western blocking buffer overnight at 4 °C. Membranes were washed as described and probed with conjugate antibodies used for ELISA at a 1:5000 dilution in Western blocking buffer for 2 h at room temperature. Membrane strips were then washed again as previously, and antibody visualised using SIGMAFAST™ 3,3′-Diaminobenzidene (DAB) tablets (Sigma Aldrich).

For the rCL1 Western blots a mouse monoclonal anti-poly-Histidine antibody (Sigma Aldrich) at a 1:500 dilution was used as the conjugate, followed by an horse raddish peroxidase (HRP) goat anti-mouse IgG (Sigma Aldrich) at 1:5000 dilution and 3,3′,5,5′-Tetramethylbenzidine (TMB) as the substrate (Sigma Aldrich).

### 2.10 2D electrophoresis

Aliquots of 150 μg of ES products were precipitated in an equal volume of 30 % trichloroacetic acid (TCA) in acetone at −20 °C overnight. Proteins were pelleted by centrifugation at 16,200 *xg* for 10 min at 4 °C and the pellet washed twice in ice-cold acetone. Pellets were dissolved in 60 μL lysis buffer (8 M Urea, 4% CHAPS, 40 mM TRIZMA base) for 1 h at room temperature. TCA precipitated ES products in lysis buffer were used to passively rehydrate non-linear 7 cm IPG Immobiline DryStrips pH 3–10 (GE Healthcare, UK) overnight. Isoelectric focussing was performed using the Ettan IPGphor II system (Amersham Biosciences). Strips were focused at 50 μA per strip at 300 V for 2.5 h (step and hold voltage mode), 1000 V for 30 min (gradient voltage mode), 5000 V for 1.5 h (gradient) and 5000 V for 1.5 h (step). Focused IPG strips were equilibrated in equilibration buffer (50 mM TRIS-Cl [pH 8.8], 6 M Urea, 30 % glycerol, 2 % SDS) containing 100 mg dithiothreitol (DTT) (VWR International, UK), followed by a second equilibration containing 250 mg iodoacetamide (IAA) (Sigma Aldrich). Proteins were separated in the second dimension using 12 % resolving SDS PAGE gels. Protein ladder was the same as used previously. Gels were stained with coomassie blue.

Immunoblotting was performed as described earlier. Serum from a naturally infected cow which had a PP of 100 and a positive FEC was used for to probe ES products. Primary serum concentration was 1:100. Secondary antibody concentration was 1:2,500.

### 2.11 In-gel trypsin digest of gel spots

Immunogenic protein spots were identified by cross referencing the immunoblot with the coomassie stained gel. Individual protein spots were carefully excised and destained in 50 mM ammonium bicarbonate and an equal volume of 50 % acetonitrile at 37 °C for 10 min. The excised spots were then reduced with 10 mM DTT for 10 min at 60 °C followed by alkylation with 55 mM IAA at room temperature for 30 min in the dark.

Gel plugs were washed in 50 mM ammonium bicarbonate and dehydrated in 100 % acetonitrile and incubated for 15 min at 37 °C. The solvent was removed, and the residual volume evaporated at 37 °C for approximately 10 min. Gel plugs were rehydrated with 0.01 μg/μl proteomic grade trypsin (Sigma Aldrich) in 50 mM ammonium bicarbonate and incubated overnight at 37 °C. Peptides were extracted using three successive 15 min incubations with 50 % (v/v) acetonitrile, 0.1 % (v/v) trifluoracetic acid (TFA). Extracts were pooled and then dried using a centrifugal evaporator (Eppendorf Concentrator Plus) and resuspended in 5 % (v/v) acetonitrile, 0.1 % (v/v) TFA.

### 2.12 NanoLC MS ESI MS/MS

Peptides were analysed by on-line Nanoflow Liquid Chromatography using the Ultimate 3000 nano system (Dionex/Thermo Fisher Scientific). Samples were first loaded onto a trap column (Acclaim PepMap 100, 2 cm x75 μm inner diameter, C18, 3 μm, 100 Å) at a flow rate of 5 μL/min with an aqueous solution containing 0.1 % (v/v) TFA and 2 % (v/v) acetonitrile. After 3 min, the trap column was set in-line an analytical column (Easy-Spray PepMap® RSLC 50 cm x75 μm inner diameter, C18, 2 μm, 100 Å) fused to a silica nano-electrospray emitter (Dionex). The column was kept at a constant 35 °C and the LC system coupled to a Q-Exactive mass spectrometer (Thermo Scientific, UK). Chromatography was performed with a buffer system consisting of 0.1 % formic acid and peptides separated by a linear gradient of 3.8–50 % buffer B (80 % acetonitrile in 0.1 % formic acid) over 30 min at a flow rate of 300 nl/min.

The Q-Exactive was kept in data-dependent mode with survey scans obtained at 70,000 at *m/z* 200. The top 10 most abundant isotope patterns with charge state +2 - +5 from the survey scan were selected from an isolation window of 2.0 Th and fragmented by higher energy collisional dissociation with normalized collision energies of 30. Maximum ion injection times for the survey scan were set at 250 ms and 100 ms MS/MS scans, with an ion target value for 1E6 for survey scans and 1E4 for MS/MS. The resolution for MS/MS events was 17,000. Repetitive sequencing of peptides was minimized by dynamic exclusion of sequenced peptides for 20 s.

### 2.13 Protein identification and quantification

Thermo RAW files were converted to mgf files using Proteowizard (version 3) (Chambers et al., 2012) and processed with the Mascot (Version 2.3.02, Matrix Science) search engine. Tandem mass spectrometry data was searched against translated Open Reading Frame’s (ORF’s) from *F. hepatica* (PRJEB25283.WBPS14, Wormbase 2019), a bespoke cathepsin database (20130227, Cwiklinski et al. (2015) plus *Bos taurus* (UniProt Ref November 2019) as background (combined 33,696 sequences; 18,233,612 residues). The search parameters were set at 10 ppm for precursor mass tolerance and 0.05 Da for fragment mass tolerance. Two missed tryptic cleavages were permitted, with carbamidomethylation (C) set as a fixed modification and oxidation (M) and deamidation (NQ) set as variable modifications.

Mascot search results were further validated using the Percolator learning algorithm within Mascot. The Mascot decoy database function was used, false discovery rate was ≤ 1% with individual percolator ion scores of more than 13 indicating identity or extensive homology (p < 0.05). Estimates of relative protein quantification within each spot was performed in Mascot to give emPAI scores (Ishihama et al., 2005). The top emPAI result for each spot was assigned to sample.

Any results not matched to the bespoke cathepsin database were manually BLAST searched against NCBI GenBank database using the protein sequence given by mass spectrometry analysis, and the top result based on E value assigned to the spot. To aid in confirmation of protein identification, the results were also searched within InterPro (Finn et al., 2017) to reveal any protein domains. Sequences were submitted to the SignalP server (Version 3.0) (Petersen et al., 2011) to identify the presence of a signal sequence and to the ExPASY Compute pI/MW tool (Gasteiger et al., 2005) to calculate theoretical pI and molecular weight of the protein. The theoretical pI and molecular weight were compared to the position of the spot within the gel.

### 2.14 Ethics statement

Blood and faecal samples were obtained from naturally and experimentally infected animals under the UK Animals Scientific (Procedures) Act 1986, licence PPL PE77BFD98. Ethical approval for collection of samples from farm animals was granted by the University of Liverpool Research Ethics Committee VREC 493 (sheep), VREC 290 and VREC 100 (cattle).

## 3 Results

### 3.1 A strong antibody response to rCL1 is detected in experimentally infected sheep and cattle

**Fig. 1:**
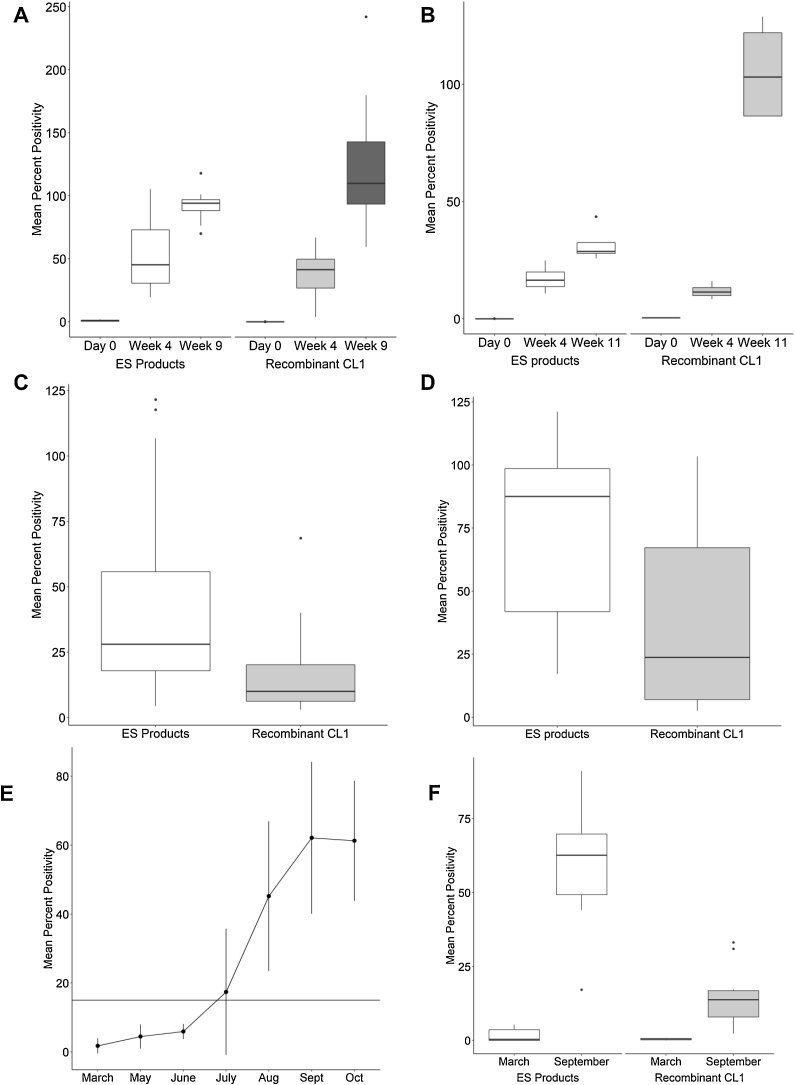
Comparisons of mean percent positivity (PP) value by *Fasciola hepatica* excretory/secretory (ES) and recombinant cathepsin L1 (rCL1) ELISAs for serum from collected from sheep (N = 12) (A) and cattle (N = 4) (B) experimentally infected with *F. hepatica* at selected time points. For 9 weeks post infection (wpi) sera from experimentally infected sheep tested against rCL1 (dark grey box), samples were diluted 1:2000 rather than 1:400. Sheep (N = 19) (C) and adult dairy cattle before drying off (N = 11), naturally infected with *F. hepatica*. (E) PP values of calves (N = 10) naturally infected with *F. hepatica* over their first year grazing season by ES ELISA. Error bars indicated +/- one standard deviation about the mean PP value. (F) comparisons of PP value by ES and rCL1 ELISA for naturally infected calves at before turn-out (March) and at housing (September). Note, y-axis scales are adjusted for the range of PP values per assay.

Sera from experimentally infected sheep (N = 12) were tested in both the ES ELISA and the rCL1 ELISA. rCL1 was successfully expressed from *P. pastoris* cultures (supplementary material 1). Sera had PP values of less than one prior to infection for both ES products and rCL1 (Fig. 1A). In the ES ELISA, the mean PP value at four wpi was 52 (standard deviation [SD] ±28) (Fig. 1A) and by nine wpi, PP values had reached a mean of 93 (SD ± 12), which remained high until the end of the infection period (14 wpi, Walsh, 2018). Sera taken at four wpi had a mean PP of 39 (SD ± 20) in the rCL1 ELISA (Fig. 1A). When sera collected at 9 wpi were tested in the rCL1 ELISA, the OD values were above the range of the ELISA reader, hence samples were retested at 1:2000 dilution (compared to 1:400 for all other time points). At this dilution, the mean PP value was 123 (SD ± 52) (Fig. 1A). All 12 sheep were FEC positive by 11 wpi.

Sera from four experimentally infected cattle (N = 4) were tested in the ES ELISA. An antibody response was detected at four wpi (Fig. 1B). At 11 wpi the mean PP was 32 (SD ± 8) and remained positive until the end timepoint (14 wpi). All cattle were FEC positive by 11 wpi. Antibodies specific for rCL1 were detected at four wpi (mean PP of 11 ± SD 3.2) (Fig. 1B). Mean PP values reached 105 PP (SD ± 22.1) by 11 wpi.

### 3.2 Naturally infected animals showed poor recognition of rCL1 by ELISA

All naturally infected adult sheep were confirmed infected by positive FEC (N = 19). Serum samples had higher mean PP values in the ES ELISA (43 PP [SD ± 37]) compared to rCL1 ELISA (15 PP [SD ± 15]) (Fig. 1C).

Sera collected from adult cattle (N = 11) from two farms were tested on the ES and rCL1 ELISA. All cattle were positive for fluke by FEC. PP values from all 11 cattle were lower against rCL1 (mean 36 PP [SD ± 34]) compared to the ES products (mean 73 PP [SD ± 33]) (Fig. 1D).

Sera collected from calves (N = 10) grazed on *F. hepatica* infected pasture over their first grazing season showed an increase in PP value by ES ELISA. All calves were negative by ES ELISA in March before turn out (mean PP < 1) (Fig. 1E). By the fifth sampling point (August), all calves were positive by ES ELISA, and remained positive (mean PP of 45 [SD ± 20]). At housing in September, PP values had reached a mean PP of 60 (SD ± 20) (Fig. 1E and F). All calves were positive by FEC by October and had not been treated. Sera from March and September were tested by rCL1 ELISA. Samples collected at September had a lower mean PP value in the rCL1 ELISA (14 PP [SD ± 10]) than ES ELISA (Fig. 1F).

### 3.3 Host antibodies from naturally and experimentally infected animals showed a different recognition of rCL1 epitopes

To identify linear B cell epitopes on the rCL1 antigen, a peptide array based on the rCL1 amino acid sequence was probed with a panel of sera from experimentally and naturally infected sheep and cattle. Our rCL1 sequence contains an extra 10 amino acids of the promoter region at the start of the sequence compared to previous published sequences (Walsh, 2018). Sera were selected that had a range of PP values in the ES and rCL1 ELISA.

**Fig. 2:**
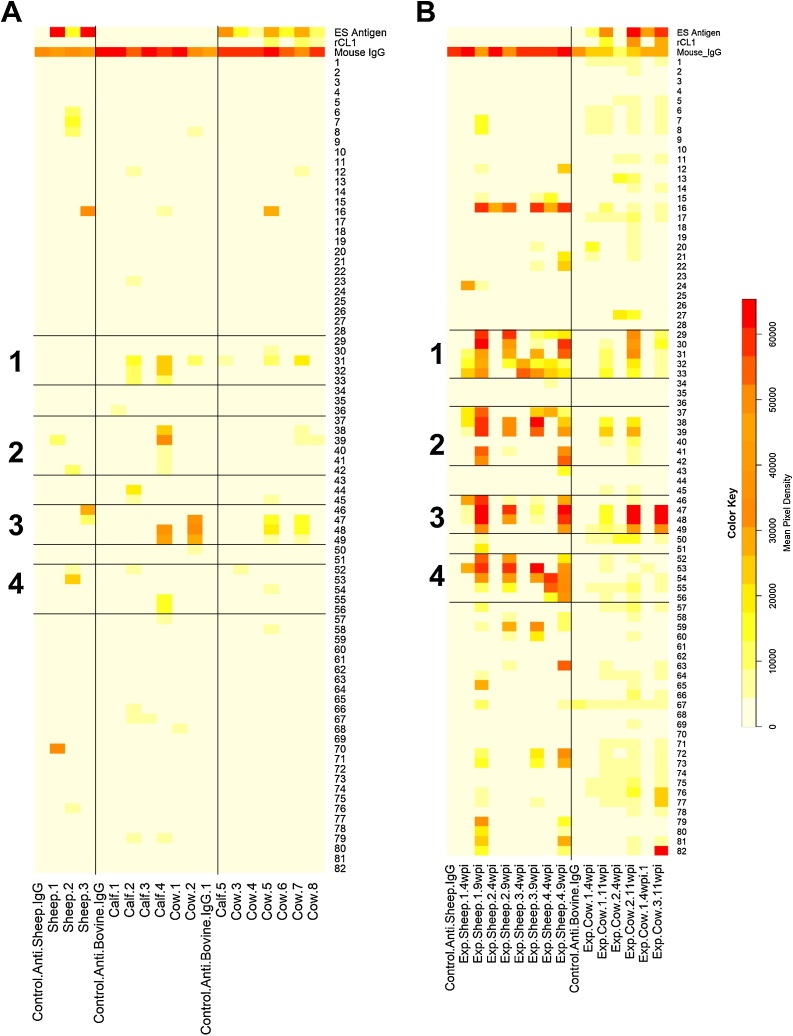
(A) Peptide array for serum from sheep experimentally infected with *Fasciola hepatica* (N = 4) at four and nine weeks post infection (wpi), and cattle experimentally infected with *F. hepatica* (N = 3) at four and 11 wpi. (B) Sheep (N = 3), calves at the September time point (N = 5) and adult cattle (N = 8) naturally infected with *F. hepatica*. Recombinant cathepsin L1 (rCL1) protein sequence is divided in peptides of 15 amino acids in length (see materials and methods). Separate arrays are indicated by the horizontal black line. Control anti-sheep and anti-bovine samples were included as shown for individual array. Intensity of colour is used to show of host antibody recognition, (represented by mean pixel density), white meaning no antibody binding, through to red which shows strong binding. Regions 1, 2, 3 and 4 with the strongest antibody recognition by experimentally infected animals are highlighted. Control protein spots of whole excretory/secretory (ES) products, rCL1 and the tertiary antibody (anti-mouse IgG) are as shown for each serum sample. For experimentally infected sheep and first naturally infected bovine array, no data is available for ES antigen and rCL1 protein spots.

**Fig. 3:**
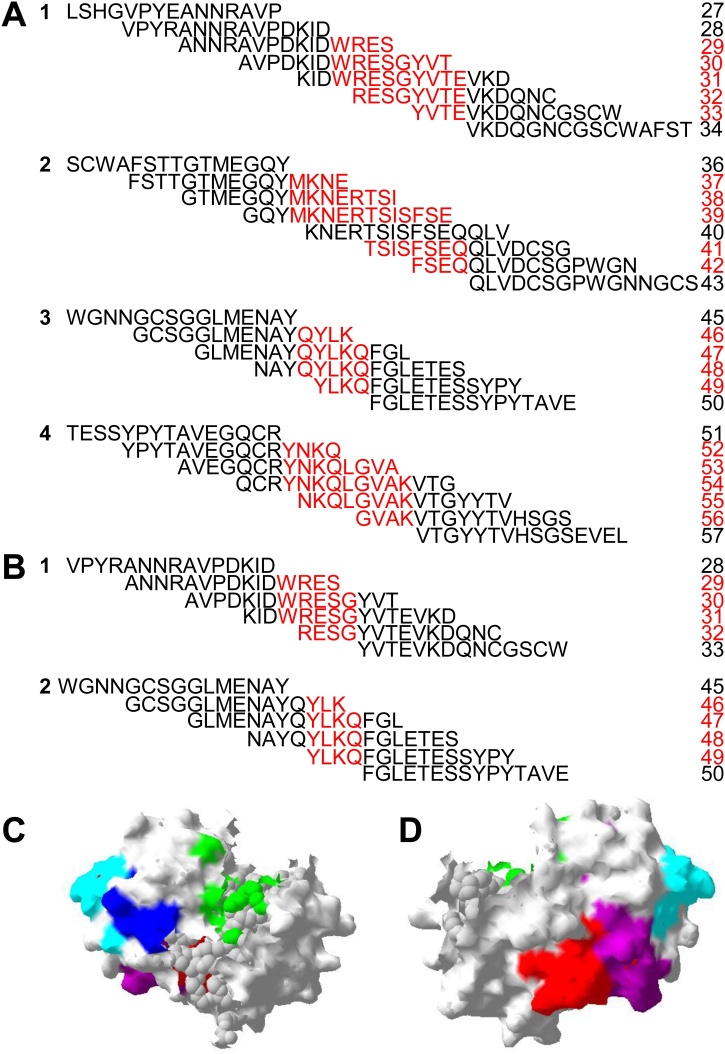
Mapping of overlapping peptides from recombinant cathepsin L1 peptide arrays which were recognised by host antibodies from sheep (A) and cattle (B) experimentally infected with *Fasciola hepatica*. The putative linear epitope is highlighted in red. Epitopes recognised by experimentally infected sheep (highlighted in red [epitope 1], purple [epitope 2], dark blue [epitope 3] and light blue [epitope 4]) were mapped onto a 3D surface model the mature CL1 protein (Stack et al., 2007) shown in (C), are located away from the active site (in green). An alternative view (rotated 180°) is shown in (D).

Sera from experimentally infected sheep showed strong recognition of rCL1 peptides (Fig. 2A). Four linear epitopes corresponding to rCL1 amino acids 124–132 (WRESGYVTE), 156–168 (MKNERTSISFSEQ), 192–196 (QYLKQ), and 216–224 (YNKQLGVAK) were recognised by sera from experimentally infected sheep (Fig. 3A). The strength of the signal was greater for sera collected 9 wpi compared to sera at 4wpi, but the overall pattern of recognition was similar (Fig. 2A). Within epitope 2 (peptides 156–168), no signal was detected to peptide 40 but there was strong binding to the overlapping consecutive peptides suggesting that the epitope covered this peptide. Sera from nine wpi recognised one peptide in the pro domain of the enzyme (peptide 16), however no other peptides were recognised suggesting this is likely to be a false positive.

Similar results were obtained with sera from experimentally infected cattle (Fig. 2A), although the overall signal was weaker. The epitopes recognised were truncated versions of two out of the four epitopes (124–128 WRESG and 193–196 YLKQ) (Fig. 3B) recognised by sera from experimentally infected sheep.

The four epitopes were mapped onto a 3D model of *F. hepatica* CL1 (Fig. 3C and D). The map shows that the epitopes are located away from the active site and accessible on the surface of the enzyme (Fig. 3C and D).

In contrast, there was little evidence for recognition of rCL1 epitopes by antibodies from naturally infected sheep or cattle (Fig. 2B). A few individual samples had variable and weak detectable signals to peptides within epitopes recognised by experimentally infected animals, but none showed recognition of the full epitope [Sheep 2 (peptide 42, 52 and 53), Sheep 3 (peptide 46 and 47), Cow 5 and Cow 7 (peptide 31, 47 and 48) and Calf 2 (peptides 30–32)] (Fig. 2B). Cow 2 had strong recognition of peptides 47–49, although weaker than the signal obtained from experimentally infected cows (Fig. 2B). Calf 4 was the most similar to experimentally infected animals, producing detectable signal to peptides (peptides 31, 32, 38, 39, 47, 48, 55 and 56) in all four of the epitopes previously identifed

### 3.4 Antibodies from experimentally infected animals and naturally infected animals recognise different proteins in ES products

**Fig. 4:**
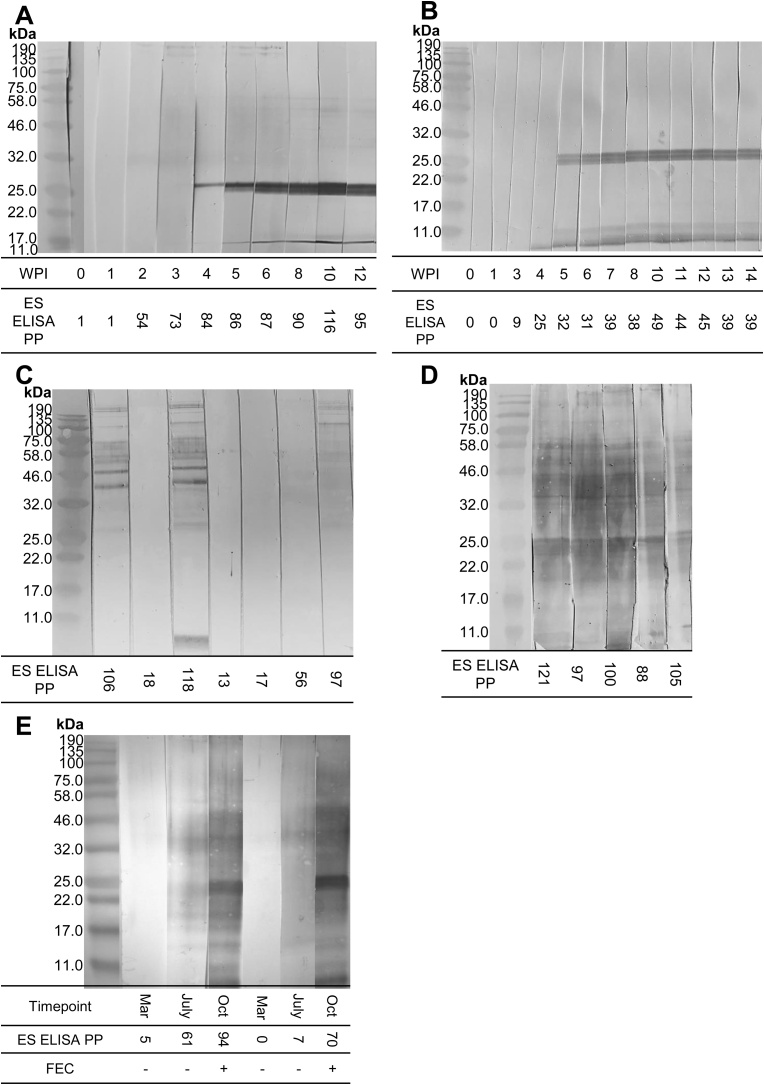
Western blots using sera collected from: one sheep (A) and one cow (B) experimentally infected with *Fasciola hepatica* at weekly time points and seven sheep (C), five adult cattle (D) and two calves (E) naturally infected with *F. hepatica*. Sera from naturally infected sheep and cattle were chosen that had a range of percent positivity (PP) values. PP values are shown below each strip.

Immunoblotting using ES products were probed with sera from experimentally infected sheep and cattle. The host response was specifically directed towards two proteins at 24−26 kDa (Fig. 4A and B) consistent with CL1 and CL2. Antibodies were detectable at four wpi in sheep and 5 wpi in cattle (Fig. 4A and B).

Immunoblotting with sera from naturally infected adult sheep, cattle and calves showed a more complex response (Fig. 4). Four sheep sera with a lower PP (<56) had no detectable signal by immunoblot. Meanwhile, sera from three naturaly infected adult sheep with high PP values (lane 2, 4 and 8) recognised a range of protein bands at <11, 25, 40, 46, 50, 58, 70, 100 and >190 kDa (Fig. 4C). Recognition of the 25 kDa protein band was weak compared to experimentally infected animals. A similar profile to adult sheep was observed with sera from five naturally infected, adult dairy cows, all of which had PP values of >88 with bands at <11, 17, 25, 35, 46, 50, 58, 75, 100 and >190 kDa (Fig. 4D). Again, strength of recognition of the 25 kDa band was variable compared to experimentally infected animals despite each cow having high PP value.

Sera from two calves collected at March (pre-turnout), July (three months post turn-out) and October (six months post-turn-out) showed an increasingly complex antibody response as exposure to natural infection developed. By October, the profile detected was similar to that observed for naturally infected adult sheep and cattle, with bands at <11, 17, 25, 35, 46, 58, 75 and ≥100 kDa (Fig. 4E).

### 3.5 Identification of ES proteins recognised by antibodies from naturally infected animals

**Fig. 5:**
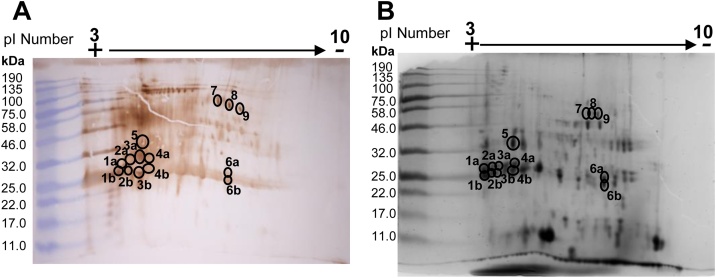
Identification of immunogenic proteins within *Fasciola hepatica* excretory/secretory (ES) products by 2D electrophoresis. (A) Western blot of 2D separated ES products probed using serum from a cow (PP 100, FEC positive) naturally infected with *F. hepatica* to identify immunogenic protein spots, (B) the corresponding coomassie stained gel annotated with immunogenic spots identified on the Western blot. Each spot is numbered and corresponds to samples used in Mass Spectrometry analysis.

ES products were separated by 2D SDS PAGE and probed with serum from a naturally infected cow (PP 100, FEC positive). Serum antibodies showed strong recognition of a string of protein spots at 25 kDa (spots 1–4 and 6), between 32−46 kDa (spot 5) and 46−58 kDa (spots 7–9) (Fig. 5A). Other protein spots were also recognised, however there was no visible corresponding protein spots on the coomassie stained gel (Fig. 5B).

The 14 spots that were most strongly recognised were analysed by mass spectrometry. The string of spots at 25 kDa (Spots 1–4 and 6, Fig. 5B) resolved as a doublet band on the coomassie stained gel, and therefore each pair was excised separately. For each pair of spots, those which resolved at higher molecular weights were denoted as ‘A’ and spots with the lower molecular weight were denoted as ‘B’.

All 14 spots showed significant matches with two or more peptides from more than one protein. The highest number of protein matches from one spot was 29. Therefore, emPAI scores were calculated for each spot. Matches were then ranked by their emPAI score and those results that gave the highest emPAI score for the spot were used as the most likely result for the spot. A summary of top protein identifications for each spot is shown in Table 1.

**Table 1:** Top putative protein identifications for each spot from 2D SDS PAGE of *Fasciola hepatica* excretory/secretory (ES) products as ranked by emPAI scores. Proteins identified through bespoke cathepsin database (Cwiklinski et al., 2015) have accession numbers based on FhCL. Proteins matched to transcripts in the Wormbase database (PRJEB25283.WBPS14) were given NCBI accession numbers.

Spot	Accession number	Description	Significant peptides matched (total)	Sequence coverage (%)	Secreted Protein (Signal IP 3.0)	Theoretical pI (ExPasy Compute pI/MW tool)	Theoretical Mass (kDa) (ExPasy Compute pI/MW tool)	emPAI score
1A	FhCL2.2	Cathepsin L2	11(15)	61	Yes	4.59	24514.02	6.61
1B	FhCL2.2	Cathepsin L2	17(18)	62.3	Yes	4.59	24514.02	95.32
2A	FhCL5	Cathepsin L5	18(24)	59.8	Yes	4.75	24468.22	47.96
2B	FhCL5	Cathepsin L5	17(23)	60.1	Yes	4.75	24468.22	40.34
3A	FhCL5	Cathepsin L5	15(22)	58.9	Yes	4.75	24468.22	8.02
3B	AAB41670.2	Cathepsin L1	14(16)	72.2	Yes	5.77	24210.17	9.34
4A	FhCL2.3	Cathepsin L2	6(6)	30.1	No	5.12	24555.38	14.84
4B	AAB41670.2	Cathepsin L1	19(20)	72.2	Yes	5.77	24210.17	405.61
5	AAB41670.2	Cathepsin L1	16(16)	70.6	Yes	5.77	24210.1	405.61
6A	P56598.2	Glutathione S-transferase	27(31)	90.5	No	6.61	25729.55	1016.13
6B	P56598.2	Glutathione S-transferase	25(34)	90.5	No	6.61	25729.55	500.76
7	THD18846.1	Dihydrolipoyl dehydrogenase mitochondrial	14(19)	56.5	No	6.19	27436.49	9.63
8	THD18846.1	Dihydrolipoyl dehydrogenase mitochondrial	11(18)	53.4	No	6.19	27436.49	8.5
9	THD18846.1	Dihydrolipoyl dehydrogenase mitochondrial	29(33)	82.2	No	6.19	27436.49	486.68

The top results from spots 1–5 were significant for *F. hepatica* cathepsin proteins for clades 1 (spots 3B, 4B & 5), 2 (spots 1A, 1B & 4A) and 5 (spot 2A, 2B & 3A). Although the top emPAI result for spot 4A was a CL2 protein, this spot also had high protein scores and significant peptide matches for a CL1 protein, matching the result for spot 4A. This could explain the slight difference in theoretical pI value and spot resolution (Fig. 5B and Table 1)

Spot 5 resolved at 32 kDa within the SDS PAGE gel, but at the same pI at spots 4A and B (Fig. 5B). The top results for this spot matched to the CL1 clade. This suggested that spot 5 was pro-CL1 and had not been cleaved to the active form.

Spot 6A and B resolved at 25 kDa, but at a higher pI value than the cathepsin proteins (Fig. 5B). The database searches for this protein gave significant matches for Glutathione S Transferase (GST) in both spots. The theoretical molecular weight and pI value calculated for these proteins correlates with the position of the spots in the gel.

Spots 7, 8 and 9 resolved at a molecular weight of approximately 46−58 kDa (Fig. 5B). These spots were highly immunogenic as shown by strong staining on the Western blot (Fig. 5A). However, in the corresponding area on the coomassie stained SDS PAGE gel only weak spots were visible, indicating their low abundance within the ES products (Fig. 5B). Results from NCBI BLAST-P search for all three spots matched to dihydrolipoyl dehydrogenase (DLD). However, the theoretical molecular weight did not correspond to the position of spots 7, 8, 9 within the gel. The emPAI scores for spot 7 and 8 (9.63 and 8.5 respectively) were lower compared to the other protein spots. For spot 9 the emPAI score was high (486.68), indicating high abundance for DLD within this spot. The high eMPAI score indicates that this identification is correct.

## 4 Discussion

Previous studies have identified that the principal immunogenic targets within *F. hepatica* ES products are the 24−26 kDa CL1 and CL2 proteases (Santiago and Hillyer, 1986; Rivera Marrero et al., 1988; Santiago and Hillyer, 1988; Coles and Rubano, 1988; Sexton et al., 1990; Smith et al., 1993a; Chauvin et al., 1995; Dalton et al., 1996; Bossaert et al., 2000; Morphew et al., 2007; Buffoni et al., 2012). These studies have used sera from experimentally infected animals given a single dose of metacercariae and collected over a 12–20 week period to demonstrate this response. In contrast there are few studies which have investigated the antibody response in naturally infected animals, which often have long-standing chronic infections from repeated exposure. In this study we showed that the responses of naturally infected animals are qualitatively different to those seen in experimentally infected animals. Naturally infected animals responded to a wide range of proteins in fluke ES products and had variable recognition of the CL1 antigen.

For commercial immunodiagnostic tests, recombinant antigens are preferred as they can be produced uniformly and in large amounts. Previously, rCL1 has been shown to be a suitable target in immunodiagnostic tests for *F. hepatica* (Alvarez Rojas et al., 2014). Consistent with these reports, we also found that sera from experimentally infected animals had strong recognition of rCL1. The earliest detectable host antibodies was at four wpi, similar to previous studies using both rCL1 (Hoyle et al., 2003; Kuerpick et al., 2013) and native ES antigens (Salimi-Bejestani et al., 2005). Conversely, samples from naturally infected animals had weaker recognition of the rCL1 antigen, compared to their response to ES products, even though all naturally infected animals had been confirmed infected through FEC and had not been treated in the six weeks prior to sampling.

Few studies have used sera from naturally infected animals to assess rCL1 antigens in diagnostic assays. Cornelissen et al. (2001) found that naturally infected cattle had antibodies that recognised a rCL1 antigen, however they did not directly compare the results to ES products. Meanwhile O’Neill et al. (1999) showed a strong correlation between OD_450_ values of human serum samples tested by rCL1 (Roche et al., 1997) and ES products. However the pattern of exposure to infection in humans is likely to differ to that in ruminants. Humans are likely to have infrequent single exposure infections, compared to repeated exposure experienced by grazing ruminants.

Peptide arrays based on our rCL1 sequence supported the results of the ELISA. These arrays showed that antibodies from experimentally infected sheep bound to four linear epitopes. Sera from experimentally infected cattle, which had lower PP values, recognised truncated versions of these same four epitopes. Sera from naturally infected animals showed poor or no recognition of the rCL1 peptides despite all but one sera sample (Sheep 1, PP 4) being above the predefined cut off for the ES ELISA (PP value range 20–121).

Mapping the four epitopes recognised by experimentally infected animals onto a 3D model of the CL1 protein showed that these eptitopes are located on the surface of the protein and away from the active site, indicating they are unlikely to prevent functional activity of the enzyme. Other studies have identified similar epitopes on CL1, using computational software (Cornelissen et al., 1999), phage display of random 12-mers peptides using rabbit anti- CL1/CL2 sera (Villa-Mancera et al., 2008, 2011; Villa-Mancera and Mendez-Mendoza, 2012; Villa-Mancera et al., 2014), or using peptide ELISAs and sera from experimentally infected cattle and sheep as part of vaccine trials (Garza-Cuartero et al., 2018; Buffoni et al., 2020). The epitopes identified in our study were in similar regions (Villa-Mancera et al., 2008, 2011; Villa-Mancera and Mendez-Mendoza, 2012; Villa-Mancera et al., 2014) but differed to epitopes identified in other studies (Garza-Cuartero et al., 2018; Buffoni et al., 2020). This is most likely to be due to the origin of the sera used. Adjuvanted vaccinations with recombinant antigens are likely to elicit a population of antibodies with a different specificity compared to a standard infection.

Differences in the peptide sequences between recombinant proteins and native antigens may result in changes to the linear B cell epitopes (Gershoni et al., 2007). Whilst our rCL1 had some variation compared to other published rCL1 protein sequences, it still had a high (93–98 %) similarity (Wijffels et al., 1994; Roche et al., 1997; Carnevale et al., 2001; Cornelissen et al., 2001). When compared to a reference sequence (Roche et al., 1997), there were no amino acid substitutions in the epitopes identified in our rCL1 sequence by peptide arrays.

Immunoblotting showed that whilst experimentally infected cattle and sheep developed highly specific responses to the CL1 and CL2 protein bands, consistent with previous studies (Santiago and Hillyer, 1986; Rivera Marrero et al., 1988; Santiago and Hillyer, 1988; Coles and Rubano, 1988; Sexton et al., 1990; Smith et al., 1993a; Chauvin et al., 1995; Dalton et al., 1996; Bossaert et al., 2000; Morphew et al., 2007; Buffoni et al., 2012), sera from naturally infected animals recognised a much wider repertoire of proteins. There was also variable recognition of the CL1 and CL2 protein bands, again consistent with the ELISA and peptide array results.

Only two other studies have described the antibody recognition of fluke ES products in naturally infected animals using immunoblotting. Gorman et al. (1997) showed that antibodies from naturally infected pigs and horses recognised a number of proteins between 40–42, 35–39, 25–30 and 14−17 kDa. Ortiz et al. (2000) used serum collected monthly over two years from naturally infected calves, heifers and adult cattle to identify immunogenic targets. Again, naturally infected cattle recognised a range of antigens in addition to the cathepsin proteins (Ortiz et al., 2000). All three groups of cattle tested recognised a group of antigens between 60−66 kDa at all time points, however only adult cattle (after first parturition) had a detectable antibody response to proteins at approximately 28 kDa which was identified by the authors as cathepsin L1 (Oblitas, 1997; Ortiz et al., 2000) and this response was variable (Oblitas, 1997). These results are consistent with the results found in this study.

To identify the antigens recognised by naturally infected animals, the protein spots which were most strongly recognised were analysed by mass spectrometry. Five proteins, CL1, CL2, CL5, GST and DLD, from 14 protein spots were identified.

*F. hepatica* show differential expression of the cathepsin Ls over different life stages (Cwiklinski et al., 2015). CL3 and CL4 are primarily expressed by newly excysted juvenile fluke, whilst CL1, CL2 and CL5 are expressed in greater amounts in adults (Cwiklinski et al., 2015). Using 2D Western blotting, we found there was strong recognition of all three adult expressed cathepsin L clades using serum from a naturally infected cow, rather than only the CL1 and CL2 as reported in previous studies (Dalton and Heffernan, 1989; Smith et al., 1993a; Dowd et al., 1994; Morphew et al., 2007).

GSTs were also recognised by serum antibodies. GST’s are primarily expressed by the parenchymal cells and are released through blebbing or shedding of the fluke tegumental surface during culture and hence are components of the ES products (Wijffels et al., 1992; Jefferies et al., 2001; Morphew et al., 2007; Robinson et al., 2009; La Course et al., 2012; Di Maggio et al., 2016). Studies using serum from experimentally infected cattle found relatively weak recognition of GSTs by serum antibodies, therefore GSTs have not been used widely in diagnostic tests although they have been tested in vaccine trials (Hillyer et al., 1992; Toet et al., 2014).

Spot 9 was identified as DLD, a mitochondrial protein and part of the pyruvate dehyrdrogenase complex (de Kok et al., 1998; Morphew et al., 2013), an important enzyme in the Krebs cycle (Tielens, 1994). This protein has not been well characterised for *F. hepatica* but is normally found in fluke tegument or somatic preparations (Morphew et al., 2013; Di Maggio et al., 2016). It is therefore likely that this protein entered the ES preparations through sloughing or damage to the tegument. We saw very strong recognition of this protein by serum antibodies from the naturally infected cow, however it has not been previously reported as a immunodiagnostic candidate. Due to the low abundance of this protein in the ES products, further work is needed to confirm if this might be a good diagnostic target for field tests.

DLD resolved into three distinct spots on the gel, indicating three potential isoforms or different post-translational modifications. Spot 9 is the most concentrated of the three spots with the highest emPAI score (486.68), suggesting that this spot is the most likely identity of the protein. We observed a discrepancy between the predicted molecular weight (27 kDa) and the resolution of the spot in the gel (approximately 48−54 kDa). An orthologue search of *F. hepatica* DLD in Wormbase Parasite indicated that matched orthologues from *Schistosoma, Clonorchis* and *Opisthorchis* were around 48−54 kDa, and the *F. hepatica* sequence was incomplete.

Our study suggests that the antibody response of animals naturally infected with *F. hepatica* may be more complex compared to the responses generated by single experimental infecton. Neither cattle or sheep acquire natural immunity to *F. hepatica* (Chauvin et al., 1995; Clery et al., 1996). Repeated infection and the long duration of most *F. hepatica* infections experienced by naturally infected animals, may result in a different population of antibodies directed at a much wider repertoire of proteins than just the CL1/CL2 antigens.

Experimentally infected animals, particulary in vaccine studies, are usually infected with a single dose of metacercariae, and then housed under controlled conditions. Most studies take place over a relatively short period of time and fluke will all mature at around the same time, between 8–10 wpi (Piacenza et al., 1999; Martínez-Fernández et al., 2004; Golden et al., 2010). In contrast, natural infections are acquired over time, so animals will be infected with a mixture of immature and mature parasites, which could survive for years in the absence of treatment (Ross, 1967). Interestingly, we found that sera from naturally infected calves, exposed to the parasite for the first time in their lives, showed strong recognition of the CL1/CL2 compared similar to experimentally infected animals. Repeated exposure to a high, continuous dose of antigens can lead to the development of a type of B cell clonal anergy known as immune paralysis, causing tolerance of the antigen (Tizard, 2008). Previous studies have shown that tolerance can develop in helminth infection due to an increase in regulatory T cells (Maizels and Mcsorley, 2016). Sachdev et al. (2017) showed that in the initial stages of chronic infection a population of CD4 T cells become hyporesponsive. These cells upregulate CTLA-4 and PD-1 but not Foxp3 (Sachdev et al., 2017). It is possible that animals which have long-lasting fluke exposure, a tolerance is generated to the cathepsins, resulting in lower antibody levels.

## 5 Conclusion

The results of this study suggest that despite the difficulty in obtaining a reliable source of native ES products, diagnostic tests utilising a combinations of antigens may be more reliable than assays based on single antigens such as CL1 for detecting animals with chronic infections. It may be possible to combine several recombinant immunodominant proteins into a cocktail of antigens to be used in a diagnostic test to provide greater diagnostic sensitivity in the field.

## Funding source

The study was supported by funding from the Agricultural and Horticultural Board (AHDB) Beef and Lamb (UK) (Grant number S2014014), the University of Liverpool and the Biotechnology and Biological Sciences Research Council, Grant number BB/K015591/1.

## Animal welfare statement

Archived serum samples from animals experimentally infected with *Fasciola hepatica* were used. Procedures were carried out under licence granted by the UK Home Office under the Animals (Scientific Procedures) Act, 1986. Archived serum samples from naturally infected sheep and cattle were used, collected by veterinarians for diagnostic purposes, with approval from the University of Liverpool Veterinary Ethics Committee.

## CRediT authorship contribution statement

**Tessa R Walsh:** Methodology, Formal analysis, Investigation, Writing - original draft, Writing - review & editing, Visualization. **Stuart Ainsworth:** Methodology, Investigation, Resources, Writing - review & editing. **Stuart Armstrong:** Methodology, Formal analysis, Investigation, Resources, Writing - review & editing. **Jane Hodgkinson:** Conceptualization, Resources, Writing - review & editing, Supervision, Project administration, Funding acquisition. **Diana Williams:** Conceptualization, Resources, Writing - review & editing, Supervision, Project administration, Funding acquisition.

## Declaration of Competing Interest

The authors declare that they have no known competing financial interests or personal relationships that could have appeared to influence the work reported in this paper.

## References

[bib0005] Alvarez Rojas C.A., Jex A.R., Gasser R.B., Scheerlinck J.P.Y. (2014). Techniques for the diagnosis of *Fasciola* infections in animals. Room for improvement. Advances in Parasitology.

[bib0010] Arnold K., Bordoli L., Kopp J., Schwede T. (2006). The SWISS-MODEL workspace: a web-based environment for protein structure homology modelling. Bioinformatics.

[bib0015] Berasaín P., Goñi F., McGonigle S., Dowd A., Dalton J.P., Frangionet B., Carmona C. (1997). Proteinases secreted by *Fasciola hepatica* degrade extracelullar matrix and basement Membrane Components. J. Parasitol..

[bib0020] Bossaert K., Farnir F., Leclipteux T., Protz M., Lonneux J.F., Losson B. (2000). Humoral immune response in calves to single-dose, trickle and challenge infections with *Fasciola hepatica*. Vet. Parasitol..

[bib0025] Bradford M.M. (1976). A rapid and sensitive method for the quantification of microgram quantities of protein utilizing the principle of protein-dye binding. Anal. Biochem..

[bib0030] Buffoni L., Martínez-Moreno F.J., Zafra R., Mendes R.E., Pérez-Écija A., Sekiya M., Mulcahy G., Pérez J., Martínez-Moreno A. (2012). Humoral immune response in goats immunised with cathepsin L1, peroxiredoxin and Sm14 antigen and experimentally challenged with *Fasciola hepatica*. Vet. Parasitol..

[bib0035] Buffoni L., Cuartero L.G., Caballero R.P., Zafra R., Moreno F.J.M., Hernández V.M., Pérez J., Moreno Á.M., Mulcahy G. (2020). Identification of protective peptides of *Fasciola hepatica* ‑ derived cathepsin L1 (FhCL1) in vaccinated sheep by a linear B ‑ cell epitope mapping approach. Parasit. Vectors.

[bib0040] Caminade C., van Dijk J., Baylis M., Williams D. (2015). Modelling recent and future climatic suitability for fasciolosis in Europe. Geospat. Health.

[bib0045] Carnevale S., Rodríguez M.I., Guarnera E.A., Carmona C., Tanos T., Angel S.O. (2001). Immunodiagnosis of fasciolosis using recombinant procathepsin L cystein proteinase. Diagn. Microbiol. Infect. Dis..

[bib0050] Chambers M.C., MacLean B., Burke R., Amodei D., Ruderman D.L., Neumann S., Gatto L., Fischer B., Pratt B., Egertson J., Hoff K., Kessner D., Tasman N., Shulman N., Frewen B., Baker T.A., Brusniak M.Y., Paulse C., Creasy D., Flashner L., Kani K., Moulding C., Seymour S.L., Nuwaysir L.M., Lefebvre B., Kuhlmann F., Roark J., Rainer P., Detlev S., Hemenway T., Huhmer A., Langridge J., Connolly B., Chadick T., Holly K., Eckels J., Deutsch E.W., Moritz R.L., Katz J.E., Agus D.B., MacCoss M., Tabb D.L., Mallick P. (2012). A cross-platform toolkit for mass spectrometry and proteomics. Nat. Biotechnol..

[bib0055] Charlier J., Duchateau L., Claerebout E., Williams D., Vercruysse J. (2007). Associations between anti-*Fasciola hepatica* antibody levels in bulk-tank milk samples and production parameters in dairy herds. Prev. Vet. Med..

[bib0060] Charlier J., Hostens M., Jacobs J., van Ranst B., Duchateau L., Vercruysse J. (2012). Integrating fasciolosis control in the dry cow management: the effect of closantel treatment on milk production. PLoS One.

[bib0065] Charlier J., Vercruysse J., Morgan E., Van Dijk J., Williams D.J.L. (2014). Recent advances in the diagnosis, impact on production and prediction of *Fasciola hepatica* in cattle. Parasitology.

[bib0070] Chauvin A., Bouvet G., Boulard C. (1995). Humoral and cellular immune responses to *Fasciola hepatica* experimental primary and secondary infection in sheep. Int. J. Parasitol..

[bib0075] Clery D., Torgerson P., Mulcahy G. (1996). Immune responses of chronically infected adult cattle to *Fasciola hepatica*. Vet. Parasitol..

[bib0080] Coles G.C., Rubano D. (1988). Antigenicity of a proteolytic enzyme of *Fasciola hepatica*. J. Helminthol..

[bib0085] Collins P.R., Stack C.M., O’Neill S.M., Doyle S., Ryan T., Brennan G.P., Mousley A., Stewart M., Maule A.G., Dalton J.P., Donnelly S. (2004). Cathepsin L1, the major protease involved in liver fluke (*Fasciola hepatica*) virulence: propeptide cleavage sites and autoactivation of the zymogen secreted from gastrodermal cells. J. Biol. Chem..

[bib0090] Cornelissen J.B.W.J., Gaasenbeek C.P.H., Boersma W., Borgsteede F.H.M., Van Milligen F.J. (1999). Use of a pre-selected epitope of cathepsin-L1 in a highly specific peptide-based immunoassay for the diagnosis of *Fasciola hepatica* infections in cattle. Int. J. Parasitol..

[bib0095] Cornelissen J.B.W.J., Gaasenbeek C.P.H., Borgsteede F.H.M., Holland W.G., Harmsen M.M., Boersma W.J.A. (2001). Early immunodiagnosis of fasciolosis in ruminants using recombinant *Fasciola hepatica* cathepsin L-like protease. Int. J. Parasitol..

[bib0100] Cwiklinski K., Dalton J.P., Dufresne P.J., La Course J., Williams D.J., Hodgkinson J., Paterson S. (2015). The *Fasciola hepatica* genome: gene duplication and polymorphism reveals adaptation to the host environment and the capacity for rapid evolution. Genome Biol..

[bib0105] Dalton J.P., Heffernan M. (1989). Thiol proteases released in vitro by *Fasciola hepatica*. Mol. Biochem. Parasitol..

[bib0110] Dalton J.P., McGonigle S., Rolph T.P. (1996). Induction of protective immunity in cattle against infection with *Fasciola hepatica* by vaccination with cathepsin L proteinases and with hemoglobin. Infect. Immun..

[bib0115] de Kok A., Hengeveld A.F., Martin A., Westphal A.H. (1998). The pyruvate dehydrogenase multi-enzyme complex from Gram-negative bacteria. Biochim. Biophys. Acta.

[bib0120] Di Maggio L.S., Tirloni L., Pinto A.F.M., Diedrich J.K., Yates J.R., Benavides U., Carmona C., da Silva Vaz I., Berasain P. (2016). Across intra-mammalian stages of the liver fluke *Fasciola hepatica*: a proteomic study. Sci. Rep..

[bib0125] Dowd A.J., Smith A.M., McGonigle S., Dalton J.P. (1994). Purification and characterisation of a second cysteine proteinase secreted by the parasitic trematode *Fasciola hepatica*. Eur. J. Biochem..

[bib0130] Finn R.D., Attwood T.K., Babbitt P.C., Bateman A., Bork P., Bridge A.J., Chang H.Y., Dosztanyi Z., El-Gebali S., Fraser M., Gough J., Haft D., Holliday G.L., Huang H., Huang X., Letunic I., Lopez R., Lu S., Marchler-Bauer A., Mi H., Mistry J., Natale D.A., Necci M., Nuka G., Orengo C.A., Park Y., Pesseat S., Piovesan D., Potter S.C., Rawlings N.D., Redaschi N., Richardson L., Rivoire C., Sangrador-Vegas A., Sigrist C., Sillitoe I., Smithers B., Squizzato S., Sutton G., Thanki N., Thomas P.D., Tosatto S.C.E., Wu C.H., Xenarios I., Yeh L.S., Young S.Y., Mitchell A.L. (2017). InterPro in 2017-beyond protein family and domain annotations. Nucleic Acids Res..

[bib0135] Fox N.J., White P.C.L., McClean C.J., Marion G., Evans A., Hutchings M.R. (2011). Predicting impacts of climate change on *Fasciola hepatica* risk. PLoS One.

[bib0140] Garza-Cuartero L., Geurden T., Mahan S.M., Hardham J.M., Dalton J.P., Mulcahy G. (2018). Antibody recognition of cathepsin L1-derived peptides in *Fasciola hepatica* -infected and/or vaccinated cattle and identification of protective linear B-cell epitopes. Vaccine.

[bib0145] Gasteiger E., Hoogland C., Gattiker A., Duvaud S., Wilkins M.R., Appel R.D., Bairoch A., Walker J.M. (2005). Protein identification and analysis tools on the ExPASy server. The Proteomics Protocols Handbook.

[bib0150] Gershoni J.M., Roitburd-Berman A., Siman-Tov D.D., Freund N.T., Weiss Y. (2007). Epitope mapping: the first step in developing epitope-based vaccines. BioDrugs.

[bib0155] Golden O., Flynn R.J., Read C., Sekiya M., Donnelly S.M., Stack C., Dalton J.P., Mulcahy G. (2010). Protection of cattle against a natural infection of *Fasciola hepatica* by vaccination with recombinant cathepsin L1 (rFhCL1). Vaccine.

[bib0160] Gorman T., Aballay J., Fredes F., Silva M., Aguillón J.C., Alcaíno H.A. (1997). Immunodiagnosis of fasciolosis in horses and pigs using Western blots. Int. J. Parasitol..

[bib0165] Gottstein B., Schneeberger M., Boubaker G., Merkle B., Huber C., Spiliotis M., Müller N., Garate T., Doherr M.G. (2014). Comparative assessment of ELISAs using recombinant saposin-like protein 2 and recombinant cathepsin L-1 from *Fasciola hepatica* for the serodiagnosis of human fasciolosis. PLoS Negl. Trop. Dis..

[bib0170] Graham-Brown J., Hartley C., Clough H., Kadioglu A., Baylis M., Williams D.J.L. (2018). Dairy heifers naturally exposed to *Fasciola hepatica* develop a type 2 immune response and concomitant suppression of leukocyte proliferation. Infect. Immun..

[bib0175] Guex N., Peitsch M.C., Schwede T. (2009). Automated comparative protein structure modeling with SWISS-MODEL and Swiss-PdbViewer: a historical perspective. Electrophoresis.

[bib0180] Hillyer G.V., de Galanes M.S., Battisti G. (1992). *Fasciola hepatica*: host responders and nonresponders to parasite glutathione S-transferase. Exp. Parasitol..

[bib0185] Howell A., Baylis M., Smith R., Pinchbeck G., Williams D. (2015). Epidemiology and impact of *Fasciola hepatica* exposure in high-yielding dairy herds. Prev. Vet. Med..

[bib0190] Hoyle D.V., Dalton J.P., Chase-Topping M., Taylor D.W. (2003). Pre-exposure of cattle to drug-abbreviated *Fasciola hepatica* infections: the effect upon subsequent challenge infection and the early immune response. Vet. Parasitol..

[bib0195] Ishihama Y., Oda Y., Tabata T., Sato T., Nagasu T., Rappsilber J., Mann M. (2005). Exponentially modified protein abundance index (emPAI) for estimation of absolute protein amount in proteomics by the number of sequenced peptides per protein. Mol. Cell Proteomics.

[bib0200] Jefferies J.R., Campbell A.M., Rossum A.J.Van, Barrett J., Brophy P.M. (2001). Proteomic analysis of *Fasciola hepatica* products. Proteomics.

[bib0205] Kamaludeen J., Graham-Brown J., Stephens N., Miller J., Howell A., Beesley N.J., Hodgkinson J., Learmount J., Williams D. (2019). Lack of efficacy of triclabendazole against *Fasciola hepatica* is present on sheep farms in three regions of England, and Wales. Vet. Rec..

[bib0210] Köstenberger K., Tichy A., Bauer K., Pless P., Wittek T. (2017). Associations between fasciolosis and milk production, and the impact of anthelmintic treatment in dairy herds. Parasitol. Res..

[bib0215] Kuerpick B., Schnieder T., Strube C. (2013). Evaluation of a recombinant cathepsin L1 ELISA and comparison with the Pourquier and ES ELISA for the detection of antibodies against *Fasciola hepatica*. Vet. Parasitol..

[bib0220] La Course E.J., Perally S., Morphew R.M., Moxon J.V., Prescott M., Dowling D.J., Neill S.M.O., Kipar A., Hetzel U., Hoey E., Zafra R. (2012). The sigma class glutathione transferase from the liver fluke *Fasciola hepatica*. PLoS Negl. Trop. Dis..

[bib0225] Li Z., Xiong F., Lin Q., d’Anjou M., Daugulis A.J., Yang D.S., Hew C.L. (2001). Low-temperature increases the yield of biologically active herring antifreeze protein in *Pichia pastoris*. Protein Expr. Purif..

[bib0230] Lowther J., Robinson M.W., Donnelly S.M., Xu W., Stack C.M., Matthews J.M., Dalton J.P. (2009). The importance of pH in regulating the function of the *Fasciola hepatica* cathepsin L1 cysteine protease. PLoS Negl. Trop. Dis..

[bib0235] Maizels R.M., Mcsorley H.J. (2016). Current perspectives Regulation of the host immune system by helminth parasites. J. Allergy Clin. Immunol..

[bib0240] Martínez-Fernández A.R., Nogal-Ruiz J.J., López-Abán J., Ramajo V., Oleaga A., Manga-González Y., Hillyer G.V., Muro A. (2004). Vaccination of mice and sheep with Fh12 FABP from *Fasciola hepatica* using the new adjuvant/immunomodulator system ADAD. Vet. Parasitol..

[bib0245] Mazeri S., Rydevik G., Handel I., Bronsvoort B.M deC., Sargison N. (2017). Estimation of the impact of *Fasciola hepatica* infection on time taken for UK beef cattle to reach slaughter weight. Sci. Rep..

[bib0250] Mezo M., González-Warleta M., Castro-Hermida J.A., Muiño L., Ubeira F.M. (2011). Association between anti-*F. hepatica* antibody levels in milk and production losses in dairy cows. Vet. Parasitol..

[bib0255] Morphew R.M., Wright H.A., LaCourse E.J., Woods D.J., Brophy P.M. (2007). Comparative proteomics of excretory-secretory proteins released by the liver fluke *Fasciola hepatica* in sheep host bile and during in vitro culture ex host. Mol. Cell Proteomics.

[bib0260] Morphew R.M., Hamilton C.M., Wright H.A., Dowling D.J., O’Neill S.M., Brophy P.M. (2013). Identification of the major proteins of an immune modulating fraction from adult *Fasciola hepatica* released by Nonidet P40. Vet. Parasitol..

[bib0265] O’Neill S.M., Parkinson M., Dowd A.J., Strauss W., Angles R., Dalton J.P. (1999). Short report: immunodiagnosis of human fascioliasis using recombinant *Fasciola hepatica* cathepsin L1 cysteine proteinase. Am. J. Trop. Med. Hyg..

[bib0270] Oblitas P.L.O. (1997). Humoral Immune Responses to *Fasciola hepatica* in Experimentally Infected Calves and in Cattle Naturally Exposed to Fasciolosis in Cajamarca.

[bib0275] Ortiz P.L., Claxton J.R., Clarkson M.J., McGarry J., Williams D.J.L. (2000). The specificity of antibody responses in cattle naturally exposed to *Fasciola hepatica*. Vet. Parasitol..

[bib0280] Ortiz P., Scarcella S., Cerna C., Rosales C., Cabrera M., Guzmán M., Lamenza P., Solana H. (2013). Resistance of *Fasciola hepatica* against Triclabendazole in cattle in Cajamarca (Peru): a clinical trial and an in vivo efficacy test in sheep. Vet. Parasitol..

[bib0285] Petersen T.N., Brunak S., Von Heijne G., Nielsen H. (2011). SignalP 4.0: discriminating signal peptides from transmembrane regions. Nat. Methods.

[bib0290] Piacenza L., Acosta D., Basmadjian I., Carmona C. (1999). Vaccination against infection with *Fasciola hepatica* in sheep with cathepsin L proteinases and with leucine aminopeptidase induce high levels of protection. Parasitol. Int..

[bib0295] Rivera Marrero C.A., Santiago N., Hillyer G.V. (1988). Evaluation of immunodiagnostic antigens in the excretory-secretory products of *Fasciola hepatica*. J. Parasitol..

[bib0300] Robinson M.W., Tort J.F., Lowther J., Donnelly S.M., Wong E., Xu W., Stack C.M., Padula M., Herbert B., Dalton J.P. (2008). Proteomics and phylogenetic analysis of the cathepsin L protease family of the helminth pathogen *Fasciola hepatica*: expansion of a repertoire of virulence-associated factors. Mol. Cell Proteomics.

[bib0305] Robinson M.W., Menon R., Donnelly S.M., Dalton J.P., Ranganathan S. (2009). An integrated transcriptomics and proteomics analysis of the secretome of the helminth pathogen *Fasciola hepatica*: proteins associated with invasion and infection of the mammalian host. Mol. Cell Proteomics.

[bib0310] Roche L., Dowd A.J., Tort J., Mcconigle S., Mcsweeney A., Curley G.P., Ryan T., Dalton J.P. (1997). Functional expression of *Fasciola hepatica* cathepsin L1 in *Saccharomyces cerevisiae*. Eur. J. Biochem..

[bib0315] Ross J.G. (1967). A comparison of the resistance status of hosts to infection with *Fasciola hepatica*. Vet. Med. Rev..

[bib0320] Sachdev D., Gough K.C., Flynn R.J. (2017). The chronic stages of bovine *Fasciola hepatica* are dominated by CD4 T-cell exhaustion. Front. Immunol..

[bib0325] Salimi-Bejestani M.R., McGarry J.W., Felstead S., Ortiz P., Akca A., Williams D.J.L. (2005). Development of an antibody-detection ELISA for *Fasciola hepatica* and its evaluation against a commercially available test. Res. Vet. Sci..

[bib0330] Salimi-Bejestani M.R., Daniel R., Cripps P., Felstead S., Williams D.J.L. (2007). Evaluation of an enzyme-linked immunosorbent assay for detection of antibodies to *Fasciola hepatica* in milk. Vet. Parasitol..

[bib0335] Santiago N., Hillyer G.V. (1986). Isolation of potential serodiagnostic *Fasciola hepatica* antigens by electroelution from polyacrylamide gels. Am. J. Trop. Med. Hyg..

[bib0340] Santiago N., Hillyer G.V. (1988). Antibody profiles by EITB and ELISA of cattle and sheep infected with *Fasciola hepatica*. J. Parasitol..

[bib0345] Sexton J.L., Milner A.R., Campbell N.J. (1990). F*asciola hepatica*: immunoprecipitation analysis of biosynthetically labelled antigens using sera from infected sheep. Parasite Immunol..

[bib0350] Smith A.M., Dowd A.J., Heffernan M., Robertson C.D., Dalton J.P. (1993). *Fasciola hepatica*: a secreted cathepsin L-like proteinase cleaves host immunoglobulin. Int. J. Parasitol..

[bib0355] Smith A.M., Dowd A.J., McGonigle S., Keegan P.S., Brennan G., Trudgett A., Dalton J.P. (1993). Purification of a cathepsin L-like proteinase secreted by adult *Fasciola hepatica*. Mol. Biochem. Parasitol..

[bib0360] Stack C.M., Donnelly S., Lowther J., Xu W., Collins P.R., Brinen L.S., Dalton J.P. (2007). The major secreted cathepsin L1 protease of the liver fluke, *Fasciola hepatica*: a Leu-12 to Pro-12 replacement in the nonconserved C-terminal region of the prosegment prevents complete enzyme autoactivation and allows definition of the molecular events in. J. Biol. Chem..

[bib0365] Stack C.M., Caffrey C.R., Donnelly S.M., Seshaadri A., Lowther J., Tort J.F., Collins P.R., Robinson M.W., Xu W., McKerrow J.H., Craik C.S., Geiger S.R., Marion R., Brinen L.S., Dalton J.P. (2008). Structural and functional relationships in the virulence-associated cathepsin L proteases of the parasitic liver fluke, *Fasciola hepatica*. J. Biol. Chem..

[bib0370] Tielens A.G. (1994). Energy generation in parasitic helminths. Parasitol.Today.

[bib0375] Tizard I.R., Tizard I.R. (2008). Regulation of acquired immunity. Veterinary Immunology - An Introduction.

[bib0380] Toet H., Piedrafita D.M., Spithill T.W. (2014). Liver fluke vaccines in ruminants: strategies, progress and future opportunities. Int. J. Parasitol..

[bib0385] Villa-Mancera A., Mendez-Mendoza M. (2012). Protection and antibody isotype responses against *Fasciola hepatica* with specific antibody to pIII-displayed peptide mimotopes of cathepsin L1 in sheep. Vet. J..

[bib0390] Villa-Mancera A., Quiroz-Romero H., Correa D., Ibarra F., Reyes-Pérez M., Reyes-Vivas H., López-Velázquez G., Gazarian K., Gazarian T., Alonso R.A. (2008). Induction of immunity in sheep to *Fasciola hepatica* with mimotopes of cathepsin L selected from a phage display library. Parasitology.

[bib0395] Villa-Mancera A., Quiroz-Romero H., Correa D., Alonso R.A. (2011). Proteolytic activity in *Fasciola hepatica* is reduced by the administration of cathepsin L mimotopes. J. Helminthol..

[bib0400] Villa-Mancera A., Reynoso-Palomar A., Utrera-Quintana F., Carreón-Luna L. (2014). Cathepsin L1 mimotopes with adjuvant Quil A induces a Th1/Th2 immune response and confers significant protection against *Fasciola hepatica* infection in goats. Parasitol. Res..

[bib0405] Walsh T. (2018). Development of a Pen-side Diagnostic Test for Liver Fluke Infections in Cattle and Sheep.

[bib0410] Wijffels G.L., Sexton J.L., Salvatore L., Pettitt J.M., Humphris D.C., Panaccio M., Spithill T.W. (1992). Primary sequence heterogeneity and tissue expression of glutathione S-transferases of *Fasciola hepatica*. Exp. Parasitol..

[bib0415] Wijffels G.L., Panaccio M., Salvatore L., Wilson L., Walker I.D., Spithill T.W. (1994). The secreted cathepsin L-like proteinases of the trematode, *Fasciola hepatica,* contain 3-hydroxyproline residues. Biochem. J..

