# Supplementary material for: Differences in the antibody response to adult Fasciola hepatica excretory/secretory products in experimentally and naturally infected cattle and sheep
Source: Vet Parasitol. 2021 Jan;289:109321. doi: 10.1016/j.vetpar.2020.109321 (PMC7840588; doi:10.1016/j.vetpar.2020.109321)
Supplement: Supplementary file 1 [file mmc1.docx]

**Supplementary material**

*Expression of rCL1 from P. pastoris*

rCL1 was successfully purified from the supernatant of transformed methanol induced *P. pastoris* cultures. rCL1 was detected in both the pro (37 kDa) and mature (25 kDa) forms (Figure S1A). Due to the low expression of rCL1 in this system (maximum yield per culture was 24 µg/ml) multiple batches were pooled and concentrated to give a final concentration of 61 µg/ml.

Antibodies in serum collected from an experimentally infected sheep at 10 wpi bound to rCL1 in a Western blot (Figure 1B). Serum from before infection (0 wpi) showed no antibody recognition (Figure S1B).


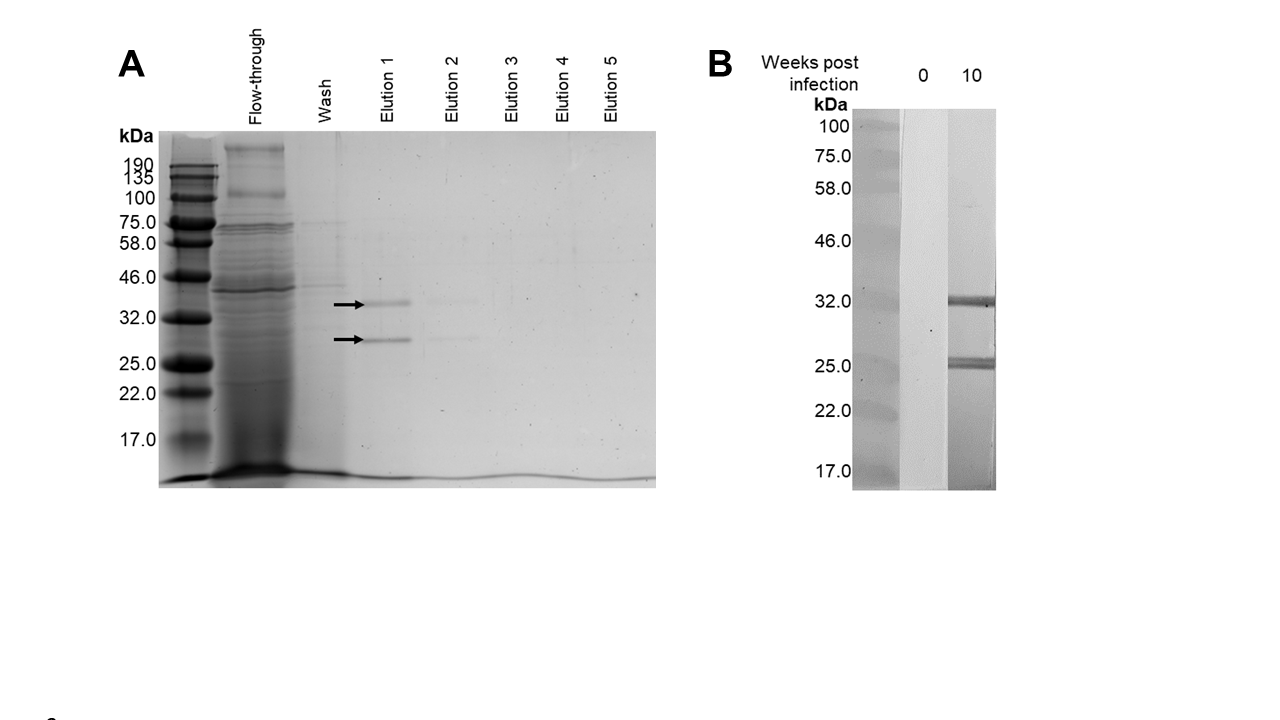
Sequence comparison using Clustal Omega (Sievers et al., 2011) showed that the expected protein sequence had 98% identity with a reference CL1 sequence (NCBI GenBank AAB41670 (Roche et al., 1997).. The rCL1 showed evidence of self activation (Collins et al., 2004; Stack et al., 2007) by SDS PAGE, indicating that it was functionally active.

Figure S1 (A) SDS PAGE of successful purification of 37 kDa pro-rCL1 and 25 kDa mature rCL1 (as indicated by the arrows) from methanol induced cultures of *P. pastoris* transformed with pPinkα-HC-CL1. (B) Western blot of purified rCL1 probed with serum from an experimentally infected sheep at 0 and 10 wpi. This sheep was FEC positive at 10 wpi and had fluke present in liver at post mortem at 16 wpi
